# Parental Declaration of Adverse Event Following Immunization in a Cross-Sectional Study in Poland

**DOI:** 10.3390/ijerph16204038

**Published:** 2019-10-22

**Authors:** Kamil Barański, Maksymilian Gajda, Bogumiła Braczkowska, Małgorzata Kowalska

**Affiliations:** Department of Epidemiology, School of Medicine in Katowice, Medical University of Silesia, 40-752 Katowice, Poland; mgajda@sum.edu.pl (M.G.); mkowalska@sum.edu.pl (M.K.)

**Keywords:** adverse event following immunization, vaccination, cross-sectional study

## Abstract

Vaccines are a well-known and effective preventive measure in communicable diseases. However, like any medical product, vaccines can cause some adverse effects. With increasing population awareness, the number of reported events related to vaccination has increased. *Aim:* The aim of the study was to assess the frequency and type of reported adverse events following childhood immunization (AEFI), and to recognize the determinant of their occurrence related with a socio-demographic situation, parental knowledge, and/or opinions on vaccinations. *Material and Methods:* The self-administrated questionnaire was distributed to a group of 3000 random parents or legal guardians living in the Silesian Voivodship (the southern part of Poland) in 2016. The response rate was eventually 41.3% from 1239 participants. Both, the number of children and the percentage of vaccinations given in the studied region, was representative for Poland as a whole. *Results:* Approximately one-third (32%) of surveyed parents declared the occurrence of AEFI in their children. The most frequently declared AEFIs were: redness, pain, swelling at the injection site (27%), and fever (24.9%). The frequency of reported AEFI was associated with a higher level of parental education and the number of vaccinations given. A negative attitude toward vaccination and the belief that vaccination is unsafe were associated with a higher number of reported AEFI. *Conclusions:* The results obtained confirmed that the number of declared mild and moderate AEFI is related to a lower parental educational level and is associated with a better experience as a consequence of a higher number of vaccinations given. Frequent AEFI reporters represent negative attitudes toward vaccinations. Further investigation with the exact surveillance system is needed to improve parental trust in vaccination safety.

## 1. Introduction

Vaccines are recognized as safe medical products. However, like any medical product, vaccines can cause some adverse events. The first vaccine for smallpox was invented in 1798 [1] while its first adverse event was reported in the 1960s [2]. With observed increased awareness, the number of reported adverse vaccine reactions (AEFI) is also increasing. Current data suggest that the number of adverse vaccine reactions in Poland ranged from 1130 cases in 2010 to 2568 cases in 2016 [3]. According to the World Health Organization (WHO) definition, an adverse vaccine reaction is the vaccine-related event caused or precipitated by a vaccine when given correctly. Adverse Event Following Immunization (AEFI) [4] is a situation where we are not fully sure that the observed adverse event is directly related to the effect of immunization. In accordance with Hill’s criteria, AEFI does not correspond with causality but has a temporal relation to immunization in parental opinion [5]. On the other hand, in Poland, any AEFI is recognized as any disorder of health status that occurred within four weeks after vaccination. The only exception is reactions after a Bacillus Calmette-Guérin (BCG) vaccination in which the temporal criterion is significantly prolonged to three months. Moreover, the Polish Ministry of Health proposes its own regulation about adverse post-vaccination reactions and criteria for their recognition. The attachment of this regulation describes the severity of adverse vaccination reactions for epidemiological surveillance. Severe AEFI is defined as a situation of required hospitalization to save health, which leads to permanent loss of physical or mental fitness or ends with death. The serious adverse reaction to a vaccination is characterized by the high severity of symptoms in the form of significant limb swelling, severe redness, and high fever but excludes all situations from severe AEFI. The weak AEFI is defined as local limb edema, strong local redness, or fever [6]. Additionally, the same attachment defines specific criteria such as local reactions, including reactions after BCG vaccination, adverse vaccination reactions on the central nervous system, and other adverse vaccination reactions.

Apart from differences resulting from the diverse definition and understanding of AEFI, some problems investigated in the current study are related to the very young age of the vaccinated children. Symptoms like fever, rash, runny nose, and irritability in children are very common in this early stage of life and can create an impression of temporary coincidence with the effect observed after vaccination. Therefore, it is very difficult to determine which of the symptoms of a health disorder is caused by the vaccination itself and which occurs independently. 

All mentioned issues are connected to the one more important factor that should be taken into account when AEFI is considered. This factor is related to the parent’s or guardian’s knowledge about currently available vaccines and vaccination programs. This likely corresponds with the socio-demographic profile of parents/guardians including their age, level of education, and financial situation. Such determinants may play a crucial role in the recognition and prevalence of reported AEFI. The aim of the study was to assess the frequency and type of reported adverse events following childhood immunization (AEFI), and recognize the determinants of their occurrence related to a socio-demographic situation, parental knowledge, and/or opinions on vaccinations.

## 2. Material and Methods

A population-based questionnaire survey was conducted in 2016, in one of the largest regions of Poland (the Silesian Voivodship) with nearly 10% of the population eligible for vaccination. Both the number of children and the percentage of vaccinations in the studied population are representative for all of Poland [7]. A total of 3000 parents or legal guardians of children aged 6–13 years from three cities (Katowice, Zabrze, and Ruda Śląska) were invited to participate in the study. Subjects were randomly selected (cluster randomization) from all 323 schools or kindergartens located in the study region. Parents were asked to fill out (anonymously and voluntarily) our own, validated questionnaire. Detailed data on the methods and tools used were presented in an earlier publication [8]. A full version of the questionnaire is available on request. In the current paper, we focus on questions related to the declaration of AEFI occurrence and their determinants related to parenteral age, level of education, and number of children in the family. The following questions gathered from the original questionnaire were used.

1. Did your child/children ever have a post-vaccination reaction after immunization? If yes, what were the symptoms?
2. Has your child/children ever had any medical contraindications for vaccinations diagnosed by a physician?
3. Do you think that qualifying tests for vaccination among children in Poland are carried out?
4. Do you think that current childhood vaccinations are safe enough?
5. Which of the following sources of knowledge about immunization is currently the source of your perception?

The Bioethical Committee of the Medical University of Silesia in Katowice, Poland (No. KNW/0022/KB/246/15) approved the study protocol. The English version of the questionnaire, which is not validated (in contrary to the native Polish version), is available upon reasonable request from the corresponding author.

### 2.1. Statistical Analysis

A chi-square test was used for assessing the diversity of AEFI occurrence among the following groups: parents with lower education vs. higher education (lower education means primary, vocational, or high school, while higher includes the other educational levels), smaller vs. larger place of residence, worse vs. better economic status of the family, declared contraindications to vaccination (yes vs. no), and evaluation of the qualification for vaccination (question number 19, categorized as good or bad). The Welch t-test conducted analysis of the mean number of vaccinations administrated per family (only children), which was classified by reporting AEFI (yes/no). The results of simple analyses were verified by binomial stepwise logistic regression predicting “any AEFI occurrence.” The detailed proportion of declared AEFI occurrence regarding the simplified categories of variables were analyzed with “do not remember” answers and transferred to the “no” answer due to its low frequency (Table 1). Missing data were excluded from all analyses and the level of significance was set at *p* < 0.05 criterion. All analyses were performed with TIBCO Software Inc. (2017) Palo Alto, USA. Statistica (data analysis software system), version 13.

## 3. Results

### 3.1. Frequency of Reported AEFI 

**Figure 1:**
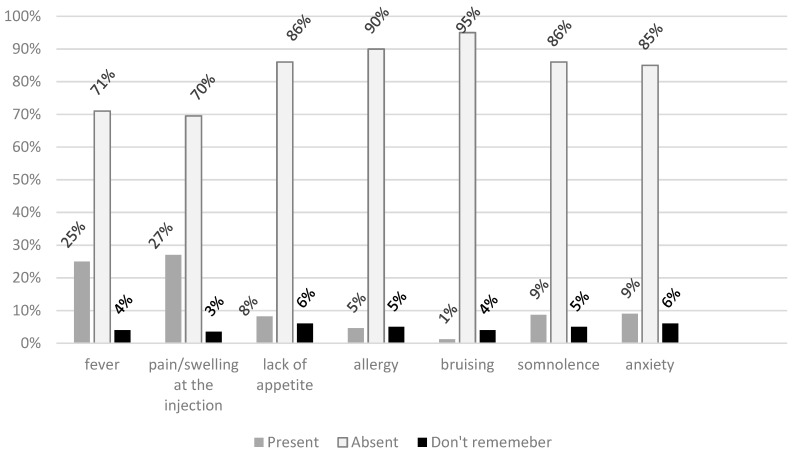
Frequency of particular reported AEFI in the question. Have your child/children ever had a post-vaccination reaction after immunization? If yes, what were the symptoms? (multi-choice-question).

Almost 32% (*N* = 394) of surveyed parents declared an occurrence of AEFI in their children, while 61% (*N* = 757) declared the absence of any AEFI and almost 6% of parents did not remember if any AFEI occurred. The most frequently mentioned AEFIs were: redness, pain, and swelling at the injection site (27.0%), and fever (25%). Less than one in ten reported: anxiety, continuous crying or screaming, somnolence, and lack of appetite (Figure 1). Detailed frequencies and numbers were presented Table S1 (supplementary materials).

### 3.2. Characteristics of Respondents and their Relation to AEFI

Mostly mothers fulfilled the questionnaire (*N* = 1083; 87%), followed by fathers (*N* = 130; 10.5%), and few legal guardians with only *N* = 15 (1.2%) (defined as people other than the biological parents). About 34% (*N* = 349) mothers and 33% (*N* = 36) fathers reported an AEFI (*p* = 0.8). More than half of respondents (*N* = 642, 51.8%) were people with a higher level of education, while 440 (35%) declared that they graduated high school, 121 (10%) graduated vocational school, and just 24 (1.9%) graduated only primary school. The majority of the respondents were urban inhabitants, where 83.3% live in largest cities and 13.5% came were from small cities. In the case of the respondents’ socioeconomic status, the results were dominated by a group of people with a very good (*N* = 746; 60.2%) or good (*N* = 280; 22.5%) financial situation. A bad situation was declared by 160 (12.9%) of the respondents, where only 25 (2%) of these were parents. The last determinant, which might influence the frequency of reported AEFI, was related to the number of children in the family. More than half of the families had two children (*N* = 654; 52.7%). Then 390 (31.4%) of the respondents had only one child, 135 (10.8%) had three children, and, lastly, 47 (3.7%) had four or more children. The detailed proportion of declared AEFI regarding the independent variables expressed by simplified categories are shown in Table 1 and detailed frequencies (in the raw form of category) were presented in Table S2 (supplementary materials).

### 3.3. Reported AEFI according to the Respondent’s Perception of Vaccination

Respondents reporting AEFIs in their children more often negatively evaluated the current Polish vaccination system (*p* < 0.001) as well as vaccinations, believing them to be unsafe (*p* < 0.001). Moreover, their children were three times more likely to have contra-indications to vaccination (*p* < 0.001). Subjects affected by the occurrence of AEFI as a child significantly more often shared false beliefs about immunization. People who experienced AEFI (compared to those who did not) more often declared that vaccinations did not affect long-term immunity (30% vs. 23%, *p* < 0.005). 

Furthermore, the parent or legal guardians who did not report any AEFI more often declared that the current vaccination program is reasonable (73% vs. 56%, *p* < 0.0001). The same no-AEFI reporters less frequently declared that vaccinations should not be performed too early (15% vs. 25%, *p* < 0.005). Moreover, the same group less frequently said that a number of vaccinations are too high and should be reduced (13% vs. 25%, *p* < 0.005). AEFI reporters more often (17%) than others (non-reporters 14%) declared improved immunity after being sick with an infectious disease is better and safer than improved immunity after vaccination in comparison to non-reporters.

The same group more often suggested that the vaccination costs outweigh the benefits (17% vs. 15%). However, the results were insignificant for both answers. 

In the following questions/opinions, (e) the realization of vaccination is indicative of parents’ concern for children’s health, (j) education in this subject is sufficient, and (k) information on the unwanted post-vaccination reactions is sufficient. No-AEFI reporters more often answered “yes” (respectively, 88% vs. 85%, 21% vs. 18%, and 29% vs. 25%; *p* = NS). Detailed results are presented in Table 2.

Multivariate analysis confirmed that reporting an AEFI might be more likely in families whose children took a high number of vaccines. (This corresponds to larger families. We excluded a number of children from the analysis since this variable is strongly correlated with the number of given vaccinations (*r* = 0.55, *p* < 0.001)). An average number of vaccines were significantly higher in families who reported AEFI in comparison to non-reporters (respectively 19.25 ± 8.86 vs. 17.25 ± 9.66; *p* = 0.0002). Moreover, the results suggest that reporting AEFI is associated with better education, with opinions about the current vaccination strategy, and that a number of given vaccinations should decrease (Table 3).

## 4. Discussion

### 4.1. Frequency of Declared AEFI and Demographic Profile of AEFI Reporters 

Most of the reported AEFIs recognized in Silesian voivodeship were mild, according to Polish regulations. Our results correspond with epidemiological data obtained for the entire country, where the frequency of mild AEFI were around 95% [9]. However, a comparison of our own data with results of other researchers from around the world confirms slightly higher values than shown in different populations. The results were around 85% to 92% for mild AEFI, depending on the study [10,11]. The meta-analysis of 36 studies conducted by Patterson et al. shows that prevalence of AEFIs ranged from 0% to 75% at the first vaccine dose against pertussis and 0%–71% at the second and third administration [12]. This indicates that almost every third or fourth child will suffer from AEFI.

In our study, the majority of AEFI reporters were female (89%), which is a high frequency in comparison with the results from Australia where females accounted for 77% [13]. When considering age groups in our study, 56% of AEFI reporters were aged between 35–44 years, which was close to the findings from the Parrella study (52.6%). Almost 60% of AEFI reporters held at least a bachelor’s degree, whereas, in Parrella’s study, only 30% of AEFI had a bachelor’s degree or higher degree. 

### 4.2. Factors Associated with Reported AEFI

The occurrence of AEFI may negatively affect parental opinion on protective vaccinations. On the other hand, the negative attitude toward vaccination might lead to exaggerated observations about symptoms that may be related to the vaccination. In our study, 64% (*N* = 763) of parents/guardians were confident about vaccine safety, 35% of parents were not sure about vaccine safety, and just four parents declared that vaccines are dangerous. The observed situation is likely related to the increasing activity of anti-vaccine movements along with unprofessional, unverified, or false information published on many Polish websites. Moreover, people cited the same findings gathered from the badly constructed, prior studies [14].

### 4.3. Surveillance and Physician’s Role in Reporting AEFI

The Polish adverse vaccine reactions’ reporting system has only a 20-year-old tradition. According to the current regulations in our country, only physicians are allowed to report AEFI. However, physicians will usually hesitate to report AEFI. First, medical doctors suggest that they are overworked and do not have enough time for one patient due to lack of assistant staff help. Second, physicians suggested that the period of time is too long between vaccinations and parental notification of AEFI. Moreover, it cannot be excluded that observed symptoms have not been related to vaccination. However, it should be strongly emphasized that a population-based active surveillance system is necessary for comparisons of rates of AEFI by vaccination status or by the time interval [15]. One of the most recognizable report systems is the Vaccine Adverse Event Reporting System (VAERS) where number of AEFI ranged from 622 to 14,532 between 1990–2018 in children and adolescents <18 years old and where a report to VAERS does not mean that healthcare staff or the vaccine caused or contributed to the adverse event [16]. VAERS registers many variables related to AEFI, but it does not include information about a person who reports AEFI. Such knowledge might be crucial in understanding the level and reasons for reported AEFI. However, it is not possible to collect socio-demographic data about AEFI reporters from a passive surveillance AEFI system. It is necessary to collect data directly from physicians who conduct immunization, since they are well educated and prepared for assessing the relationship between a particular vaccination and potential AEFI [17]. Moreover, such a role can be fulfilled by nurses since they have received formal training in vaccine safety and reporting as medical practitioners [18]. However, in the study conducted by Gorman’s team, there is a suggestion that migrants do not believe health visitors because, in respondents’ opinions, they are not well prepared for their role [19].

### 4.4. Limitations of the Study

The limitations of our study are very similar to the limitations found elsewhere [5]. The first that should be mentioned is the type of study. Untruthful answers are inevitably part of a cross-sectional study and they are difficult to verify. We were not able to verify our data with medical records because of the nature of the study (our own questionnaire was anonymous). Another limitation is related to the time point when we conducted the study. All parents were fulfilling the questionnaire after at least one session of vaccination. We believe that such a study should be performed in a group of people who have no children. Another limitation is linked to the calendar of the compulsory vaccination program. However, our study was conducted in a short time period, which might decrease a possible random error. The last important limitation of the presented study was related to the variables that we have not measured. The health status of vaccinated children could be helpful information in deciding whether symptoms occurred after vaccination or if it was related to earlier symptoms of the disease. We believe that all mentioned limitations, despite their quantity, were reduced by selecting a representative group, which is the strongest value of the study. 

Lastly, it is necessary to remind that an AEFI is any untoward medical occurrence, which follows immunization and which will not necessarily have a causal relationship with administration of the vaccine. The adverse event may be any unfavorable or unintended sign, or any abnormal laboratory finding, symptom, or disease [4].

## 5. Conclusions

There is no doubt that the AEFIs are common, but they are not serious and not unexpected. Parents’ or guardians’ educational levels play a key role in frequency and types of reported AEFI (at least mild AEFI). The Polish surveillance system should be a component of passive and active collection data about AEFI where a team of healthcare staff with a physician or qualified nurse as a leader should validate the reported AEFI. 

## Figures and Tables

**Table 1:** The detailed proportion of declared AEFI occurrence regarding the simplified categories of variables *.

Variable	Value	Level of Education			Number of Children			Place of Residence			Financial Situation		
		Lower	Higher	*p*	One	More	*p*	Small City	Big City	*p*	Bad	Better	*p*
		*N* = 597	*N* = 642		*N* = 390	*N* = 849		*N* = 168	*N* = 1071		*N* = 25	*N* = 1214	
Any AEFI	Yes	162; 27.1%	232; 36.1%	**	98; 25.2%	296; 34.9%	**	57; 34.0	337; 31.5	NS	7; 28.0	387; 31.9	NS
	No	435; 72.9%	410; 63.9%		292; 74.8%	553; 65.1%		111; 66.0	734; 68.5		18; 72.0	808; 68.1	
Fever	Yes	132; 22.1	177; 27.6	**	74; 19.0	235; 27.7	**	52; 31.0	257; 24.0	NS	4; 16.0	305; 25.1	NS
	No	465; 77.9	465; 72.4		316; 81.0	614; 72.3		116; 69.0	814; 76.0		21; 84.0	909; 74.9	
At the injection	Yes	143; 23.9	192; 29.9	**	83; 21.3	252; 29.7	**	47; 28.0	288; 26.9	NS	6; 24.0	329; 27.1	NS
	No	454; 76.1	450; 70,1		307; 78.7	597; 70.3		121; 72.0	783; 73.1		19; 76.0	885; 72.9	
Lack of appetite	Yes	45; 7.5	57; 8.9	NS	17; 4.4	85; 10.0	**	10; 5.9	92; 8.6	NS	4; 16.0	98; 8.1	NS
	No	552; 92.5	585; 91.1		373; 95.6	764; 90.0		158; 94.1	979; 91.4		21; 84.0	91.9	
Allergy	Yes	21; 3.5	36; 5.6	NS	11; 2.8	46; 5.4	**	10; 5.9	47; 4.4	NS	0	57; 4.7	N/A
	No	576; 96.5	606; 94.4		379; 97.2	803; 95.6		158; 94.1	1024; 95.6		25	1157; 95.3	
Bruising	Yes	11; 1.8	4; 0.6	NS, F	1; 0.2	14; 1.6	** F	3; 1.8	12; 1.1	NS	0	15; 1.2	N/A
	No	586; 98.2	638; 99.4		389; 99.8	835; 98.4		165; 98.2	1059; 98.9		25	1199; 98.8	
Somnolence	Yes	54; 9.0	54; 8.4	NS	18; 4,6	90; 10.6	**	13; 7.7	95; 8.9	NS	2; 8.0	106; 8.7	NS
	No	543; 91.0	588; 91.6		372; 95.4	759; 89.4		155; 92.3	976; 91.1		23; 92.0	1108; 91.3	
Anxiety	Yes	48; 8.0	64; 10.0	NS	25; 6.4	87; 10.2	**	16; 9.5	96; 9.0	NS	2; 8.0	110; 9.0	NS
	No	549; 92.0	578; 90.0		365; 93.6	762; 89.8		152; 90.5	975; 91.0		23; 92.0	1104; 91.0	

* All analyses were performed after excluding missing variables for all specific reported AEFI. ** *p* < 0.05, NS—not significant in Chi-square or in Fisher test (F), N/A—statistics not available; AEFI - Adverse events following immunization.

**Table 2:** Parental opinion according to the reported adverse event following immunization.

Parental Opinion *N* = (1220)		AEFI (Adverse Event Following Immunization)		
		Yes	No	*p*
		*N* = 394	*N* = 826	
a. Vaccinations are a very important method for the prevention of infectious diseases	Yes	360	771	NS
	No	34	55	
b. The evidence of vaccinations’ efficacy is insufficient	Yes	97	238	NS
	No	297	588	
c. Vaccinations did not provide long-term immunity	Yes	119	196	**
	No	275	630	
d. Being sick with an infectious disease results in better immunity than vaccination	Yes	66	120	NS
	No	328	706	
e. The realization of vaccination is indicative of parents’ concern for children’s health	Yes	337	726	NS
	No	57	100	
f. The current vaccination strategy is reasonable	Yes	223	604	***
	No	171	222	
g. Vaccination should not be performed too early	Yes	101	123	***
	No	293	703	
h. The number of vaccinations is too high and should be reduced	Yes	100	111	***
	No	294	715	
i. The vaccination costs outweigh the benefits	Yes	66	121	NS
	No	328	795	
j. Education in this subject is sufficient	Yes	72	173	NS
	No	322	653	
k. Information on the unwanted post-vaccination reactions is sufficient	Yes	97	238	NS
	No	297	588	

Legend: * <0.05, *** <0.005, NS—nonsignificant, *p*-value in Chi-square test or Fisher test used as appropriate.

**Table 3:** Stepwise logistic regression model for AEFI prediction (*n* = 1219).

Parameter		Odds Ratio Estimates		
		Point Estimates	Adjusted OR (95% CI)	*p* Value
**Number of vaccines (*n*)**	quantitative variable	1.026	1.01–1.04	<0.001
Education level	lower vs. higher	0.723	0.56–0.93	0.01
Vaccination are safe	yes vs. no	0.554	0.42–0.73	<0.001
Current vaccination strategy is reasonable	yes vs. no	0.640	0.48–0.84	0.001
The number of vaccines is too high and should be decreased	yes vs. no	1.648	1.18–2.88	0.002

## Supplementary Materials

The following are available online at https://www.mdpi.com/1660-4601/16/20/4038/s1 **Table S1**. Any adverse effects following the immunization (AEFI) regarding specific types of reported AEFI. Table S2. The detailed proportion of declared AEFI regarding the variables from metrics.
