# Supplementary material for: Parental Declaration of Adverse Event Following Immunization in a Cross-Sectional Study in Poland
Source: Int J Environ Res Public Health. 2019 Oct 22;16(20):4038. doi: 10.3390/ijerph16204038 (PMC6843965; doi:10.3390/ijerph16204038)
Supplement: Supplementary file 1 [file ijerph-16-04038-s001.pdf]

Table S1. Any adverse effects following the immunization (AEFI) regarding specific types of reported AEFI.

Occurrence of AEFI % (N)	Any AEFI	Fever	at the Injection Site	Lack of Appetite	Allergy	Bruising	Somnolence	Anxiety
Present	31.8% (394)	24.9% (309)	27.0% (335)	8.2% (102)	4.6% (57)	1.2% (15)	8.7% (108)	9.0% (112)
Absent	61.1% (757)	71.2% (882)	69.5% (861)	85.7% (1062)	90.2% (1117)	94.7% (1173)	85.7% (1062)	85.3% (1157)
Don't remember	5.6% (69)	3.9% (48)	3.5% (43)	6.1% (75)	5.2% (65)	4.1% (51)	5.6% (69)	5.6% (70)

Table S2. The detailed proportion of declared AEFI regarding the variables from metrics.

Variable		Fulfilled by		Educational Level				Place of Residence		Number of Children in the Family			
		Mother N; %	Father N; %	Primary N; %	Vocational N; %	Highschool N; %	Higher education N; %	Small city N; %	Big city N; %	1 N; %	2 N; %	3 N; %	4 N; %
Fever	Yes	277; 25.6	25; 19.2	8; 33.3	29; 24.0	91; 20.7	177; 27.6	52; 30.9	243; 23.5	74; 19.0	174; 26.6	43; 31.8	16; 34.0
	No	806; 74.4	105; 80.8	16; 66.7	92; 76.0	349; 79.3	465; 72.4	116; 69.1	790; 76.5	316; 81.0	480; 73.4	92; 68.2	31; 66.0
At the injection	Yes	292; 27.0	33; 50.2	6; 25.0	24; 19.8	108; 24.5	192; 29.9	47; 28.0	274; 26.5	83; 21.3	189; 28.9	45; 33.3	15; 31.9
	No	791; 73.0	32; 49.8	18; 75.0	114; 94.2	332; 75.5	450; 70.1	121; 72.0	759; 73.5	307; 78.7	465; 71.1	90; 66.7	32; 68.1
Lack of appetite	Yes	89; 8.2	11; 8.5	6; 25.0	7; 5.8	30; 6.8	57; 8.8	10; 6.0	87; 8.4	17; 4.3	61; 9.3	16; 11.8	7; 14.9
	No	994; 91.8	119; 91.5	18; 75.0	114; 84.2	410; 93.2	585; 91.2	158; 94.0	946; 91.6	373; 95.7	593; 90.7	119; 88.2	40; 85.1
Allergy	Yes	46; 4.2	9; 6.9	1; 4.2	2; 1.6	16; 3.6	36; 5.6	10; 6.0	44; 4.3	11; 2.8	35; 5.3	6; 4.4	4; 8.5
	No	1037; 95.8	121; 93.1	23; 95.8	119; 98.4	424; 96.4	606; 94.4	158; 94.0	989; 95.7	379; 97.2	619; 94.7	129; 95.6	43; 91.5
Bruising	Yes	13; 1.2	1; 0.7	0	2; 1.6	8; 1.8	4; 0.6	3;1.8	12; 1.1	1; 0.2	10; 1.5	2; 1.5	2; 4.3
	No	1070; 98.2	129; 99.3	24; 100	119; 98.4	432; 98.2	638; 99.4	165; 98.2	1021; 98.9	389; 99.8	664 98.5	133; 98.5	45; 95.7

Somnolence	Yes	94; 8.7	11; 8.5	4; 16.7	13; 10.7	36; 8.2	54; 8.4	13; 7.7	92; 8.9	18; 4.6	65; 9.9	19; 14.1	5; 10.6
	No	989; 91.3	119; 91.5	20; 83.3	108; 89.3	404; 91.8	588; 91.6	152; 90.5	941; 91.1	372; 95.4	589; 90.1	116; 85.9	42; 89.4
Anxiety	Yes	101; 9.3	9; 6.9	3;12.5	7; 5.8	37; 8.4	64; 10.0	16; 9.5	91; 8.8	25; 6.4	61; 9.3	18; 13.3	7; 14.9
	No	982; 90.7	121; 93,1	21; 87.5	114; 84.2	403; 91.6	578; 90.0	152; 90.5	942; 91.2	365; 93.6	593; 90.7	117; 86.7	40; 85.1
